# Association of Maternal Citizenship and State-Level Immigrant Policies With Health Insurance Coverage Among US-Born Latino Youths

**DOI:** 10.1001/jamanetworkopen.2020.21876

**Published:** 2020-10-21

**Authors:** Cinthya K. Alberto, Jessie Kemmick Pintor, Maria-Elena Young, Loni Philip Tabb, Ana Martínez-Donate, Brent A. Langellier, Jim P. Stimpson

**Affiliations:** 1 Dornsife School of Public Health, Drexel University, Philadelphia, Pennsylvania; 2 Department of Public Health, School of Social Sciences, Humanities and Arts, University of California, Merced

## Abstract

**Importance:**

The association of state-level immigrant policies with uninsurance among Latino youths remains unknown.

**Objective:**

To assess the association of state-level immigrant integration and criminalization policies with health insurance coverage among US-born Latino youths by maternal citizenship.

**Design, Setting, and Participants:**

This cross-sectional study analyzed secondary data from the American Community Survey, January 1, 2016, to December 31, 2018, for US-born Latino youths (age, ≤17 years) and their mothers (age, 18-64 years) as well as state-level indicators of immigrant integration and criminalization policies (in all 50 states and the District of Columbia).

**Exposures:**

Immigrant integration and criminalization policies.

**Main Outcomes and Measures:**

The main outcome was maternal reports of youth uninsurance status at the time of the American Community Survey interview. Variation in youth uninsurance by maternal citizenship, state immigrant integration policy context, and state immigrant criminalization policy context were examined. All analyses were conducted with weighted survey data.

**Results:**

Of the 226 691 US-born Latino youths (115 431 [50.92%] male; mean [SD] age, 7.66 [4.92] years) included in the study, 36.64% (95% CI, 36.21%-36.92%) had noncitizen mothers. Overall, 7.09% (95% CI, 6.78%-7.41%) of noncitizen mothers reported that their youths were uninsured compared with 4.68% (95% CI, 4.49%-4.88%) of citizen mothers. Of uninsured youths who resided in states with a low level of immigrant integration policies, 9.10% (8.22%-10.06%) had noncitizen mothers and 4.75% (95% CI, 4.19%-5.37%) had citizen mothers; of uninsured youths who resided in states with high criminalization policies, 9.37% (95% CI, 8.90%-9.87%) had noncitizen mothers and 5.91% (95% CI, 5.64%-6.20%) had citizen mothers. In states with few immigrant integration policies, the probability of uninsurance among youths with noncitizen mothers was 3.3% (95% CI, 2.3%-4.4%) higher than that among youths with citizen mothers. Among youths with noncitizen mothers, the difference in the probability of uninsurance between those residing in states with a low level vs a high level of immigrant integration policies was 2.1% (95% CI, 0.6%-3.6%). Among youths residing in states with high levels of immigrant criminalization policies, those with noncitizen mothers had a 2.6% (95% CI, 1.9%-3.0%) higher probability of being uninsured compared with those whose mothers were citizens. Among youths with noncitizen mothers, the difference in the probability of uninsurance between those who resided in a state with a low level vs a state with a high level of immigrant criminalization policies was 1.7% (95% CI, 0.7%-2.7%).

**Conclusions and Relevance:**

The findings of this cross-sectional study suggest that among US-born Latino youths, disparities in uninsurance by maternal citizenship are associated with state-level immigrant integration and criminalization policies and that anti-immigrant policies may be associated with disparities in health care access for US-born Latino youths.

## Key Points

**Question:**

Are state-level policies that integrate or criminalize immigrants associated with disparities in youth health insurance coverage by maternal citizenship status?

**Findings:**

In this cross-sectional study of 226 691 US-born Latino youths, residing in a state with a lower level of immigrant integration policies and a higher level of immigrant criminalization policies was associated with higher uninsurance rates. Uninsurance rates for youths with non–US citizen mothers were higher for those who resided in states with low integration and high criminalization immigrant policies.

**Meaning:**

State-level immigrant policies may be associated with health care access among US-born Latino youths based on maternal citizenship status, and awareness of these disparities appears to be needed to inform advocacy efforts.

## Teaser

This cross-sectional study uses data from the American Community Survey to examine the association of state-level immigrant integration and criminalization policies and maternal citizenship status with health insurance coverage among US-born Latino youths.

## Introduction

Latino youths comprise nearly a quarter of all youths in the US,^1^ and approximately 94% of Latino youths were born in the US.^2^ Although Latino youths account for the largest ethnic minority group among youths in the US,^1^ health insurance coverage is lower among Latino youths (92%) than among non-Latino White (96%), African American (95%), and Asian (95%) youths.^3^ Parental immigration and citizenship status have been associated with insurance coverage for Latino youths.^4,5,6^ Latino youths whose parents are citizens have higher rates of health insurance coverage compared with Latino youths whose parents are mixed status,^7^ noncitizen,^8,9^ or non-US born.^10,11^ Even among youths whose immigrant parents have health insurance coverage, youths with noncitizen parents are more likely to be uninsured.^7,8,12^

Some of the disparities in insurance coverage, health care, and health among US-born Latino youths likely are associated with structural factors and policies that make it difficult for their immigrant parents to gain insurance coverage for themselves.^13,14^ Because of their citizenship status, many immigrant parents are ineligible for public insurance programs, including the Patient Protection and Affordable Care Act Medicaid expansion, further exacerbating disparities in access to care for immigrant families and their US-born youths.^15,16,17^ Noncitizen Latina mothers who work for employers who do not offer benefits are more likely to be uninsured,^18,19^ and thus, their US-born Latino youths are more likely to also be uninsured and have decreased odds of receiving annual well-child visits and preventive care.^20^ Seeking health care access and services for US-born Latino youths may be a barrier for Latina immigrant mothers owing to language, discrimination, and challenges in navigating the health care system.^21^ Overall, these systematic challenges are associated with health care access, utilization, and overall health for US-born Latino youths.^22^

State-level immigrant policies are influential structural factors that facilitate or hinder access to certain benefits.^16,23,24,25^ Some state-level policies explicitly exclude noncitizens through their alignment with federal-level immigration policies that exclude individuals from resources, such as subsidies for health insurance, or eligibility for programs, such as Medicaid, based on their legal status.^15,26,27,28^ Integration policies can facilitate access to programs for immigrant populations, and criminalization policies can increase risk of exposure to law enforcement, detention, and deportation.^29^ State-level integration policies include access to photo identification and driver’s licenses, Medicaid coverage for those who have resided in the state for less than 5 years, access to health insurance for children regardless of legal status, wage and hour protections for workers, and laws protecting noncitizen workers from employer retaliation related to their legal status.^23,29^ State-level criminalization policies include state compliance with Real ID (a federal law that requires states to implement uniform standards for issuance of identification and driver’s licenses) and a state mandate for employers to use E-Verify (an internet system that confirms eligibility of employees to work in the US).^23,29^ Immigrant integration and criminalization policies can have direct and indirect association with health care access for immigrant families and their US-born youths.^25,30,31^ Exclusionary immigrant policies, heightened immigration law enforcement, and anti-Latino rhetoric have been found to be associated with a reduction in health insurance uptake for US-citizen youths of Latina immigrant mothers.^32,33,34^ The association of maternal citizenship and state-level immigrant integration and criminalization policies with uninsurance for US-born Latino youths is unknown.

The association of immigration policy with the overall health of Latino immigrant populations in the US has been documented. Arrests, deportations, and raids by US Immigration and Customs Enforcement^35^ and racist rhetoric during and after the 2016 presidential election have been associated with preterm births and low-birth-weight infants born to Latina mothers in the US.^36,37^ Using the Andersen Behavioral Model of Health Services^38^ framework encompassing demographic and income factors, we examined variation in uninsurance by state-level integration and criminalization immigrant policies for US-born Latino youths with citizen and noncitizen mothers. Given that state-level immigrant integration and criminalization policies are forms of legalized discrimination based on legal status,^29^ it is important to examine variation by maternal citizenship status. We hypothesized that uninsurance would be more common among youths with noncitizen mothers and among youths who reside in states that have the fewest immigrant integration policies and the most immigrant criminalization policies.

## Methods

### Data Source

This cross-sectional study used pooled American Community Survey (ACS) data from January 1, 2016, to December 31, 2018, from the Integrated Public Use Microdata Series.^39^ These data are publicly available and de-identified, and thus, the Drexel University institutional review board exempted this study from human subjects protocol review. This study followed the Strengthening the Reporting of Observational Studies in Epidemiology (STROBE) reporting guideline.^40^

The ACS is a yearly nationwide survey administered by the US Census Bureau and collects information on demographic, social, and economic characteristics. It is mailed to approximately 295 000 US addresses per month, or 3.5 million households per year, in all 50 US states, the District of Columbia, and Puerto Rico. Response rates were 94.7% in 2016, 93.7% in 2017, and 92% in 2018.^41^ The ACS contains state-level Federal Information Processing Standard codes, and Latino ethnicity is self-reported in the ACS.^42^ The study sample consisted of Latino youths (17 years or younger) for whom linked Latina maternal characteristics (eg, citizenship status) were available. We only included youths of Latina mothers who were younger than 65 years at the time of the survey and who reported that their youngest child was no older than 17 years at the time of the survey. The sample size after applying the inclusion criteria was 226 691.

### Measures

Youth uninsurance was the outcome measure of interest and was assessed based on the mothers’ reports of whether their youths had any health insurance coverage at the time of the survey (yes or no). The main independent variables were maternal citizenship status (US citizen or noncitizen), 1 binary measure of state-level immigrant integration policy (high integration or low integration), and 1 binary measure of state-level immigrant criminalization policy (low criminalization or high criminalization). We classified state-level immigrant integration and criminalization policies using the Young index of state-level immigrant integration and criminalization policy context.^29^ The index was created through a systematic review of implemented immigrant policies up to December 31, 2015, and policies were classified as integration or criminalization. Fourteen policies were classified as integration because they either facilitated or contributed to barriers for immigrant access to state institutions and programs (eg, access to health insurance coverage for children regardless of legal status, extension of wage and hour protections for agricultural workers and domestic workers, and laws protecting noncitizen workers from employer retaliation related to their legal status).^29^ The criminalization category included 6 policies that could influence activities of noncitizens by increasing or decreasing their exposure to law enforcement (eg, complying with Real ID, mandating employer use of E-Verify, and requiring or allowing law enforcement to verify an individual’s legal status during an arrest or traffic stop). A table listing all of these policies can be found in eTable 1 in the Supplement.

The total numbers of integration and criminalization policies were summed separately, and median and mean scores of immigrant policy contexts were calculated. We used the median score to classify states and the District of Columbia as high integration or low integration and as low criminalization or high criminalization (based on whether they were at or above the median vs below the median). In general, states were in the high-integration category if they had 4 or more integration policies and in the low-integration category if they had 3 or fewer integration policies. The high-criminalization category included states with 4 or more criminalizing policies, and the low-criminalization category included states with 3 or fewer criminalizing policies.^29^ Summaries of integration and criminalization policies and integration and criminalization categorizations by state are presented in eTables 2 and 3 in the Supplement.

We used the Andersen Behavioral Model of Health Services^38^ to select control variables in the models. Factors included youth age (0-4, 5-9, 10-13, or 14-17 years) and sex (male or female) and mother’s language (English or Spanish), marital status (married, divorced or separated, or never married), age (18-29, 30-39, 40-49, or 50-64 years), number of birth children (1-3 or ≥4), employment status (working or with a job, unemployed, or not in the labor force), and educational level (less than high school diploma, high school diploma, or college degree or more). We adjusted for income as a percentage of the federal poverty level (≥400%, 300%-399%, 200%-299%, 100%-199%, or ≤99%). We controlled for contextual characteristics from the 2016 presidential election by creating a binary indicator of states whose electoral college voted for Hillary Clinton or voted for Donald Trump.^43^ We also included survey year (2016, 2017, and 2018) and state (all 50 states and the District of Columbia) fixed effects.

### Statistical Analysis

We conducted a secondary data analysis of ACS data using Stata, version 15 (StataCorp LLC).^44^ First, we used descriptive statistics to examine differences in youth uninsurance and among the aforementioned demographic and socioeconomic factors by maternal citizenship status using χ^2^ tests. Second, we used χ^2^ tests to examine variation in youth uninsurance by maternal citizenship (citizen or noncitizen), state immigrant integration policy context (low integration or high integration), and state immigrant criminalization policy context (high criminalization or low criminalization). Third, initial multivariable logistic regressions were used to assess whether associations existed among maternal citizenship status and youth uninsurance while controlling for the demographic and socioeconomic variables. We added state integration and criminalization policy variables into the models and then tested interactions between maternal citizenship and state integration and criminalization policy when assessing youth uninsurance. Comparisons and associations were considered significant at 2-sided *P* < .05. In addition, we calculated the probability of youth uninsurance by running a margins command to examine the mean marginal association of maternal citizenship and of state integration and criminalization policy with youth uninsurance. We also used sensitivity models to exclude states that covered prenatal care regardless of documentation status; states that used a 4-level, state-level immigrant policy measure that combined integration and criminalization; and states with continuous measures of integration and criminalization (eTables 7-10 in the Supplement). All analyses were conducted with weighted survey data to produce estimates of the noninstitutionalized population of US-born Latino youths.

## Results

Of 226 691 US-born Latino youths (115 431 [50.92%] male; mean [SD] age, 7.66 [4.92] years), 63.40% (63.02%-63.89%) had citizen mothers and 36.64% (95% CI, 36.21%-36.92%) had noncitizen mothers. A total of 5.56% (95% CI, 5.40%-5.74%) of youths were uninsured, and 94.44% (95% CI, 94.26%-94.60%) were insured; 7.09% (95% CI, 6.78%-7.41%) of noncitizen mothers reported youth uninsurance compared with 4.68% (95% CI, 4.49%-4.88%) of citizen mothers. Overall, 97.38% (95% CI, 97.17%-97.58%) of noncitizen mothers reported speaking Spanish as their primary language compared with 70.10% (95% CI, 69.67%-70.53%) of citizen mothers, and 53.84% (95% CI, 53.17%-54.52%) of noncitizen mothers reported an educational level less than a high school diploma compared with 16.37% (95% CI, 15.99%-16.76%) of citizen mothers (Table 1). Higher proportions of noncitizen mothers compared with citizen mothers resided in low integration states (16.66% [95% CI, 16.21%-17.17%] vs 12.93% [95% CI, 12.67%-13.21%]) as well as in low criminalization states (43.71% [95% CI, 43.12%-44.31%] vs 40.23% [95% CI, 39.86%-40.59%]).

**Table 1.** Sample Characteristics Among US-Born Latino Youths and Latina Mothers by Maternal Citizenship Status^a^

Characteristic	Total, weighted mean % (95% CI) (N = 226 691)	Maternal citizenship status, weighted mean % (95% CI)		*P* value^**b**^
		US citizen (n = 143 722)	Noncitizen (n = 82 969)	
Individuals	100	63.40 (63.02-63.89)	36.64 (36.21-36.92)	NA
Youth uninsurance				
No	94.44 (94.26-94.60)	95.32 (95.12-95.51)	92.91 (92.59-93.22)	<.001
Yes	5.56 (5.40-5.74)	4.68 (4.49-4.88)	7.09 (6.78-7.41)	
Youth age, y				
0-4	32.27 (32.02-32.53)	33.22 (32.90-33.55)	30.63 (30.22-31.05)	<.001
5-9	30.43 (30.21-30.65)	29.80 (29.52-30.07)	31.53 (31.16-31.90)	
10-13	22.00 (21.80-22.20)	21.25 (21.00-21.50)	23.30 (22.96-23.64)	
14-17	15.30 (15.11-15.49)	15.73 (15.50-15.97)	14.55 (14.25-14.85)	
Female youths	49.08 (48.83-49.33)	49.13 (48.82-49.45)	48.98 (48.55-49.41)	.57
Mother’s primary language				
English	19.91 (19.62-20.21)	29.90 (29.47-30.33)	2.62 (2.42-2.83)	<.001
Spanish	80.09 (79.79-80.38)	70.10 (69.67-70.53)	97.38 (97.17-97.58)	
Maternal marital status				
Married	63.06 (62.68-63.44)	60.86 (60.39-61.33)	66.88 (66.24-67.52)	.16
Divorced or separated	11.51 (11.26-11.77)	13.25 (12.92-13.58)	8.51 (8.13-8.91)	
Never married	25.42 (25.08-25.77)	25.89 (25.48-26.32)	24.61 (24.03-25.19)	
Maternal age, y				
18-29	24.38 (24.04-24.71)	27.36 (26.93-27.78)	19.21 (18.69-19.74)	.25
30-39	52.09 (51.70-52.48)	49.59 (49.11-50.08)	56.42 (55.74-57.08)	
40-49	20.69 (20.38-20.99)	19.97 (19.61-20.33)	21.93 (21.39-22.48)	
50-64	2.85 (2.75-2.95)	3.08 (2.96-3.21)	2.44 (2.28-2.61)	
Maternal youths, No.				
1-3	81.60 (81.22-81.97)	83.98 (83.53-84.43)	77.46 (76.78-78.13)	<.001
≥4	18.40 (18.03-18.78)	16.02 (15.57-16.47)	22.54 (21.87-23.22)	
Maternal employment				
Working or has a job	57.85 (57.46-58.24)	66.12 (65.65-66.58)	43.52 (42.85-44.20)	<.001
Unemployed	4.54 (4.38-4.71)	4.78 (4.57-5.00)	4.12 (3.87-4.40)	
Not in labor force	37.61 (37.22-38.00)	29.10 (28.65-29.55)	52.35 (51.67-53.03)	
Maternal educational level				
Less than high school diploma	30.08 (29.71-30.46)	16.37 (15.99-16.76)	53.84 (53.17-54.52)	<.001
High school diploma	54.39 (54.00-54.79)	62.57 (62.11-63.03)	40.22 (39.56-40.89)	
College degree or more	15.52 (15.27-15.77)	21.06 (20.70-21.41)	5.94 (5.65-6.22)	
State immigrant integration policy level				
High integration	85.70 (85.50-85.90)	87.07 (86.79-87.33)	83.34 (82.87-83.79)	<.001
Low integration	14.30 (14.10-14.50)	12.93 (12.67-13.21)	16.66 (16.21-17.17)	
State immigrant criminalization policy level				
Low criminalization	41.50 (41.24-41.76)	40.23 (39.86-40.59)	43.71 (43.12-44.31)	<.001
High criminalization	58.50 (58.24-58.76)	59.77 (59.41-60.14)	56.29 (55.69-56.88)	
Income, % FPL				
≥400%	13.56 (13.34-13.79)	18.87 (18.55-19.20)	4.37 (4.13-4.61)	<.001
300%-399%	9.14 (8.93-9.35)	11.56 (11.28-11.86)	4.94 (4.68-5.22)	
200%-299%	16.86 (16.58-17.15)	18.74 (18.37-19.12)	13.60 (13.16-14.05)	
100%-199%	31.07 (30.70-31.44)	27.16 (26.73-27.61)	37.83 (37.17-38.50)	
≤99%	29.37 (28.99-29.74)	23.66 (23.22-24.10)	39.26 (38.58-39.94)	
2016 Electoral college result				
Hillary Clinton	51.52 (51.26-51.78)	50.57 (50.19-50.95)	53.16 (52.56-53.77)	<.001
Donald Trump	48.48 (48.22-48.74)	49.43 (49.05-49.81)	46.84 (46.23-47.44)	
Survey year				
2016	33.26 (32.89-33.64)	32.56 (32.11-33.02)	34.47 (33.83-35.12)	<.001
2017	33.42 (33.04-33.80)	33.42 (32.97-33.88)	33.41 (32.77-34.07)	
2018	33.32 (32.94-33.70)	34.02 (33.56-34.48)	32.12 (31.47-32.77)	

Abbreviations: FPL, federal poverty level; NA, not applicable.

^a^ Data are from the Integrated Public Use Microdata Series of the American Community Survey, January 1, 2016, to December 31, 2018.

^b^ *P* values are based on χ^2^ tests.

Differences in the proportion of youth uninsurance by state immigrant integration and criminalization policy contexts and maternal citizenship are presented in Table 2. Of youths in high integration states, 5.39% (95% CI, 5.22%-5.57%) were uninsured compared with 6.60% (95% CI, 6.11%-7.14%) in low integration states. The percentage of uninsured youth with noncitizen mothers was 6.69% (95% CI, 6.36%-7.03%) in high integration states compared with 9.10% (95% CI, 8.22%-10.06%) in low integration states. In high criminalization states, the percentage of uninsured youths with citizen mothers was 5.91% (95% CI, 5.64%-6.20%). Of youths with noncitizen mothers, 4.15% (95% CI, 3.80%-4.54%) in low criminalization states were uninsured compared with 9.37% (95% CI, 8.90%-9.87%) in high criminalization states.

**Table 2.** Youth Uninsurance Percentage by State Immigrant Policy Context and Maternal Citizenship^a^

State immigrant policy context	Youth uninsurance, weighted % (95% CI)		*P* value^**b**^
	No (n = 213 996)	Yes (n = 12 695)	
**Integration**			
High integration			
Citizen mother	95.33 (95.12-95.53)	4.67 (4.47-4.88)	NA
Noncitizen mother	93.31 (92.97-93.64)	6.69 (6.36-7.03)	NA
Total	94.61 (94.43-94.78)	5.39 (5.22-5.57)	<.001
Low integration			
Citizen mother	95.25 (94.63-95.81)	4.75 (4.19-5.37)	NA
Noncitizen mother	90.90 (89.94-91.78)	9.10 (8.22-10.06)	NA
Total	93.40 (92.86-93.89)	6.60 (6.11-7.14)	<.001
**Criminalization**			
Low criminalization			
Citizen mother	97.15 (96.9-97.37)	2.85 (2.63-3.10)	NA
Noncitizen mother	95.85 (95.46-96.20)	4.15 (3.80-4.54)	NA
Total	96.65 (96.44-96.84)	3.35 (3.16-3.56)	<.001
High criminalization			
Citizen mother	94.09 (93.80-94.36)	5.91 (5.64-6.20)	NA
Noncitizen mother	90.63 (90.13-91.10)	9.37 (8.90-9.87)	NA
Total	92.87 (92.61-93.12)	7.13 (6.88-7.39)	<.001

Abbreviation: NA, not applicable.

^a^ Data are from the Integrated Public Use Microdata Series of the American Community Survey, January 1, 2016, to December 31, 2018.

^b^ *P* values are based on χ^2^ tests.

Table 3 presents the probabilities and pairwise comparisons of youth uninsurance by maternal citizenship and state immigrant policy environments from multivariate logistic regression. The probability of youth uninsurance in the high level of integration immigrant state-level policy context was 1.3% (95% CI, 0.3%-2.2%; *P* < .05) higher for youths with noncitizen mothers compared with youths with citizen mothers. Compared with youths with citizen mothers in low integration state-level policy contexts, the probability of youth uninsurance was 3.3% (95% CI, 2.3%-4.4%; *P* < .001) higher for youths with noncitizen mothers. The youth uninsurance rate was 2.1% (95% CI, 0.6%-3.6%; *P* = .006) higher among youths with noncitizen mothers in low integration states compared with youths with noncitizen mothers in high integration states. There was no difference between youths with noncitizen and citizen mothers in the probability of youth uninsurance in states with low levels of criminalization policies. Conversely, in high criminalization states, there was a 2.6% (95% CI, 1.9%-3.0%; *P* < .001) higher probability of uninsurance among youths with noncitizen mothers compared with youths with citizen mothers. The probability of youth uninsurance among youths with noncitizen mothers in states with high criminalization policy contexts was 1.7% (95% CI, 0.7%-2.7%; *P* < .001) higher than that in states with low criminalization policy contexts. The full regression and probabilities for all citizenship and policy context results are presented in eTables 4-5 in the Supplement. Results were similar to those of the sensitivity analyses performed (eTables 7-10 in the Supplement).

**Table 3.** Probabilities and Pairwise Comparisons of Youth Uninsurance by Latina Maternal Citizenship Status and State Immigrant Policy Context Based on Logistic Regression Models^a^

State immigrant policy context	Probability of youth uninsurance (95% CI)	*P* value
**Integration context**		
Maternal citizenship		
US citizen	1 [Reference]	NA
Noncitizen and high integration	0.013 (0.003 to 0.022)	<.05
Noncitizen and low integration	0.033 (0.023 to 0.044)	<.001
Pairwise comparison	0.021 (0.006 to 0.036)	.006
**Criminalization context**		
Maternal citizenship		
US citizen	1 [Reference]	NA
Noncitizen and low criminalization	0.008 (−0.001 to 0.016)	.07
Noncitizen and high criminalization	0.026 (0.019 to 0.030)	<.001
Pairwise comparison	0.017 (0.007 to 0.027)	<.001

Abbreviation: NA, not applicable.

^a^ Data are from the Integrated Public Use Microdata Series of the American Community Survey, January 1, 2016, to December 31, 2018. Models were adjusted for youth age and sex; maternal language, marital status, age, number of youths, employment status, educational level, and income; results of the 2016 presidential election; survey year; and state.

## Discussion

Latino youths who are US born and have immigrant parents face persistent barriers in access to health care compared with youths whose parents are US natives.^4,5,6^ Some of these disparities are associated with parental uninsurance disparities owing to differences in documentation and citizenship status that can impact eligibility for programs and access to affordable benefits offered by employers.^7,8,12,15,16,18^ State-level immigrant policies may be associated with a greater ability for immigrants to access health care. For instance, California has allowed young adult undocumented immigrants to access the state’s Medicaid program.^45^ We sought to understand the associations among maternal citizenship, state-level immigrant integration and criminalization policies, and US-born Latino youth uninsurance. We observed that immigrant integration policies were not associated with uninsurance among youths with citizen mothers but that a higher proportion of uninsurance among youths with noncitizen mothers was associated with a context of low levels of immigrant integration policy. High levels of immigrant criminalization policy were associated with uninsurance among US-born Latino youths; in this study, almost 6% of youths who had citizen mothers and lived in high criminalization states were uninsured.

Disparities in insurance coverage among US-born Latino youths may limit the ability of this group, the largest ethnic minority youth group in the US, to receive annual well-child visits and preventive care.^20^ We observed that in high integration states, uninsurance rates were 1.3% higher for youths with noncitizen mothers compared with youths whose mothers were citizens. In contrast, in low integration states, uninsurance rates were 3.3% higher for youths with noncitizen mothers compared with youths whose mothers were citizens. The uninsurance rate of 1.3% equated to approximately 232 700 uninsured youths in the US. In contrast, in low integration states, uninsurance rates were 3.3% higher for youths with noncitizen mothers compared with youth with citizen mothers, equating to approximately 590 700 youths in the US. On average, US-born Latino youths with noncitizen mothers had a 2.1% higher probability of being uninsured in a low integration state compared with living in a high integration state. Translating this 2.1% probability into quantities, an estimated 375 900 US-born Latino youths with noncitizen mothers in the US were uninsured, and this was associated with state-level integration policies.^46^ High levels of immigrant integration policies at the state level were associated with lower uninsurance for youths with noncitizen mothers; thus, these policies may be associated with reduced disparities in access to care for US-born Latino youths. Low levels of immigrant integration policies may be associated with less ability of immigrants, including parents of US-born Latino youths, to integrate into programs and benefits, which by extension may have consequences for US-born youths who may be entitled to these programs and benefits. From a health policy and cost–benefit perspective, providing health insurance coverage for uninsured youths, specifically youths eligible for Medicaid or the Children’s Health Insurance Program, could save the US $8 billion to $10 billion annually.^47^

High levels of immigrant criminalization policies at the state level were significantly associated with a 2.6% higher probability of uninsurance for youths with noncitizen mothers compared with youths whose mothers were citizens. These findings suggest that noncitizen parents, particularly mothers, may face structural barriers to seeking health care access, programs, and benefits for their US-born youths. Fear of detention and/or deportation that arises from residing in a state with a high level of immigrant criminalization policies may be associated with uninsurance disparities for US-born Latino youths and also has been found to discourage immigrant mothers from seeking well-child visits for their US-born infants.^48,49^ State-level criminalization policies may exacerbate social and economic disparities among Latino immigrant communities. For instance, requiring all employers to use E-Verify excludes immigrants based on their documentation and citizenship status from employment opportunities where they may have been offered benefits including health insurance coverage.^18^ Employers in these states may exploit immigrant populations that are disadvantaged owing to their status, because employers can threaten employees if they vocalize any violations of their rights (eg, working conditions, wages, and sick leave).^50^

### Limitations

This study has limitations. We relied on a multidimensional index that measured whether certain state-level policies were enacted as of December 31, 2015, which limited our ability to use a preimplementation–postimplementation study design. We relied on ACS data from 2016 to 2018 to examine the association between state policies and uninsurance of Latino youths, assuming that state-level classifications in the index did not drastically shift, such as from high to low integration or low to high criminalization. Although this is a limitation, using this index was important because it measured multiple immigrant policies that capture state-level immigrant integration and criminalization policy contexts that might be associated with uninsurance among US-born Latino youth. To our knowledge, this approach has not been done previously. Second, we could not assess whether there have been any changes to the probability of uninsurance for the same youths over time. Third, the estimates in this study may be conservative given that participating in the ACS may be discouraging for noncitizen mothers in the current sociopolitical climate. Fourth, there may be other factors across states that could be associated with health insurance but were not measured in this study and were assumed to be held constant by the state and year fixed-effects analysis. In addition, analyses should be conducted to elucidate whether state-level policies for immigrant integration and criminalization are associated with other factors such as income and employment for immigrant parents and their US-born youths, which in turn may be associated with health care access, utilization, and outcomes over time.

## Conclusions

In this cross-sectional study, disparities in youth uninsurance were associated with state-level policies and contexts for integration and criminalization of immigrants. Overall, state-level anti-immigration policies may have negative spillover effects on health care access for Latino youths who are US citizens and who may be entitled to programs such as Medicaid and/or the Children’s Health Insurance Program. In turn, Latino youths may continue to experience barriers to accessing and receiving well-child visits and preventive care visits.

## Notes

Supplement. **eTable 1.** Number of state immigrant criminalization and integration policies, enacted by December 31, 2015 **eTable 2.** State immigrant criminalization and integration policies, enacted by December 31, 2015 **eTable 3.** Categorization of US states and the District of Columbia by state-level integration and criminalizing policies **eTable 4.** Odds ratios from logistic regression estimation of youth uninsurance by Latina maternal citizenship status and state immigrant policy environments **eTable 5.** Predicted probability of youth uninsurance by Latina maternal citizenship status and integration state-level immigrant policy context **eTable 6.** Predicted probability of youth uninsurance by Latina maternal citizenship status and criminalization state-level immigrant policy context **eTable 7.** Predicted probabilities and pairwise comparison of predicted probabilities of youth uninsurance by Latina maternal citizenship status and state immigrant policy environments, excluding states who covered prenatal care regardless of documentation status **eTable 8.** Predicted probabilities and pairwise comparison of youth uninsurance by Latina maternal citizenship status and 4-level state-level immigrant policy measure that combined integration and criminalization **eTable 9.** Predicted probabilities of youth uninsurance by Latina maternal citizenship status and continuous integration state-level immigrant policy context summation **eTable 10.** Predicted probabilities of youth uninsurance by Latina maternal citizenship status and continuous criminalization state-level immigrant policy context
