# Supplementary material for: Association of Maternal Citizenship and State-Level Immigrant Policies With Health Insurance Coverage Among US-Born Latino Youths
Source: JAMA Netw Open. 2020 Oct 21;3(10):e2021876. doi: 10.1001/jamanetworkopen.2020.21876 (PMC7578764; doi:10.1001/jamanetworkopen.2020.21876)
Supplement: Supplement. — eTable 1. Number of state immigrant criminalization and integration policies, enacted by December 31, 2015 eTable 2. State immigrant criminalization and integration policies, enacted by December 31, 2015 eTable 3. Categorization of US states and the District of Columbia by state-level integration and criminalizing policies eTable 4. Odds ratios from logistic regression estimation of youth uninsurance by Latina maternal citizenship status and state immigrant policy environments eTable 5. Predicted probability of youth uninsurance by Latina maternal citizenship status and integration state-level immigrant policy context eTable 6. Predicted probability of youth uninsurance by Latina maternal citizenship status and criminalization state-level immigrant policy context eTable 7. Predicted probabilities and pairwise comparison of predicted probabilities of youth uninsurance by Latina maternal citizenship status and state immigrant policy environments, excluding states who covered prenatal care regardless of documentation status eTable 8. Predicted probabilities and pairwise comparison of youth uninsurance by Latina maternal citizenship status and 4-level state-level immigrant policy measure that combined integration and criminalization eTable 9. Predicted probabilities of youth uninsurance by Latina maternal citizenship status and continuous integration state-level immigrant policy context summation eTable 10. Predicted probabilities of youth uninsurance by Latina maternal citizenship status and continuous criminalization state-level immigrant policy context [file jamanetwopen-e2021876-s001.pdf]

Supplementary Online Content

Alberto CK, Kemmick Pintor J, Young ME, et al. Association of maternal citizenship and state-level immigrant policies with health insurance coverage among US-born Latino youths. *JAMA Netw Open*. 2020;3(10):e2021876. doi:10.1001/jamanetworkopen.2020.21876

eTable 1. Number of state immigrant criminalization and integration policies, enacted by December 31, 2015
eTable 2. State immigrant criminalization and integration policies, enacted by December 31, 2015
eTable 3. Categorization of US states and the District of Columbia by state-level integration and criminalizing policies
eTable 4. Odds ratios from logistic regression estimation of youth uninsurance by Latina maternal citizenship status and state immigrant policy environments
eTable 5. Predicted probability of youth uninsurance by Latina maternal citizenship status and integration state-level immigrant policy context
eTable 6. Predicted probability of youth uninsurance by Latina maternal citizenship status and criminalization state-level immigrant policy context
eTable 7. Predicted probabilities and pairwise comparison of predicted probabilities of youth uninsurance by Latina maternal citizenship status and state immigrant policy environments, excluding states who covered prenatal care regardless of documentation status
eTable 8. Predicted probabilities and pairwise comparison of youth uninsurance by Latina maternal citizenship status and 4-level state-level immigrant policy measure that combined integration and criminalization
eTable 9. Predicted probabilities of youth uninsurance by Latina maternal citizenship status and continuous integration state-level immigrant policy context summation
eTable 10. Predicted probabilities of youth uninsurance by Latina maternal citizenship status and continuous criminalization state-level immigrant policy context

This supplementary material has been provided by the authors to give readers additional information about their work.

© 2020 Alberto et al. *JAMA Network Open*.

eTable 1. Number of state immigrant criminalization and integration policies, enacted by December 31, 2015.		
**State**	**Integration Policies**	**Criminalization Policies**
Alabama	2	6
Alaska	2	3
Arizona	5	5
Arkansas	3	3
California	12	1
Colorado	7	2
Connecticut	7	2
Delaware	3	3
District of Columbia	9	2
Florida	6	5
Georgia	2	4
Hawaii	7	2
Idaho	3	4
Illinois	8	1
Indiana	3	5
Iowa	4	4
Kansas	3	4
Kentucky	3	4
Louisiana	3	4
Maine	6	3
Maryland	6	3
Massachusetts	6	4

© 2020 Alberto et al. *JAMA Network Open*.

Michigan	7	4
Minnesota	9	4
**State**	**Integration Policies**	**Criminalization Policies**
Mississippi	2	5
Missouri	3	4
Montana	5	3
Nebraska	4	4
Nevada	5	2
New Hampshire	3	3
New Jersey	5	4
New Mexico	5	3
New York	11	4
North Carolina	3	5
North Dakota	5	3
Ohio	3	4
Oklahoma	4	4
Oregon	7	3
Pennsylvania	2	4
Rhode Island	6	3
South Carolina	3	4
South Dakota	4	3
Tennessee	3	5
Texas	8	5
Utah	4	3
Vermont	5	2
Virginia	2	4
Washington	8	1

© 2020 Alberto et al. *JAMA Network Open*.

West Virginia	4	5
Wisconsin	6	4
**State**	**Integration Policies**	**Criminalization Policies**
Wyoming	3	4

Source: Young M-EDT, Wallace SP. Included, but Deportable: A New Public Health Approach to Policies That Criminalize and Integrate Immigrants. American Journal of Public Health. 2019;109(9):1171-1176.

© 2020 Alberto et al. *JAMA Network Open*.

eTable 2. State immigrant criminalization and integration policies, enacted by December 31, 2015.

Criminalization Policies	Integration Policies
State drivers licenses (Does the state require a social security number to obtain a driver's license?)	State Children's Health Insurance Program (SCHIP) (Does the state provide health insurance to children regardless of legal status?)
Compliance with federal Real ID Act of 2005, sets standards for state licenses ID's (Does the state comply with Real ID?)	Medicaid—prenatal care (Does the state provide care to pregnant women regardless of legal status?)
Use of E-Verify (Does state mandate employers use of E-Verify?)	Supplemental Nutrition Assistance Program (Does the state count a prorated share of ineligible noncitizen income to determine family eligibility for benefits?)
Law enforcement collaboration with federal enforcement (Does state fully collaborate with federal immigration authorities?)	In-state college and university tuition (Does the state provide most students in-state tuition regardless of legal status?)
Law enforcement inquiry about legal status (Does the state require or allow that law enforcement verify individuals' legal status at the time of a stop or arrest?)	Financial aid for colleges and universities (Does the state provide students scholarships or financial aid regardless of legal status?)
Sentencing laws (Does the state sentence nonviolent criminal offenses at least 365 d?)	Citizenship requirements for peace officers (Does the state require peace officers be citizens?)
	Citizenship requirements for teachers (Does the state require teachers be citizens?)
	Workers compensation (Does the state include undocumented immigrants in the definition of employee?)
	Extension of protections for agricultural (Does the state extend wage and hour protections for agricultural workers?)

© 2020 Alberto et al. *JAMA Network Open*.

Criminalization Policies	Integration Policies
	Extension of protections for domestic workers (Does the state extend wage and hour protections for domestic workers?)
	Domestic Worker's Bill of Rights (Does the state have a Domestic Worker's Bill of Rights?)
	Protection against immigration-related employer retaliation (Does the state have laws that protect noncitizen workers from employer retaliation related to their legal status?)
	Professional licensing of undocumented and DACAmented professionals (Does the state allow licensing of undocumented or DACAmented professionals?)
	Payment of interpreters through Medicaid or SCHIP (Does the state pay for interpreters through Medicaid or SCHIP?)
	English language–only legislation (Does the state have English as the official language?)
Source: Young M-EDT, Wallace SP. Included, but Deportable: A New Public Health Approach to Policies That Criminalize and Integrate Immigrants. American Journal of Public Health. 2019;109(9):1171-1176.	

© 2020 Alberto et al. *JAMA Network Open*.

**eTable 3. Categorization of US states and the District of Columbia by state-level integration and criminalizing policies.**			
**Integration**		**Criminalization**	
**High Integration**	**Low Integration**	**Low Criminalization**	**High Criminalization**
Arizona	Alabama	Alaska	Alabama
Florida	Georgia	Arkansas	Georgia
Iowa	Idaho	Delaware	Idaho
Massachusetts	Indiana	New Hampshire	Indiana
Michigan	Kansas	California	Kansas
Minnesota	Kentucky	Colorado	Kentucky
Nebraska	Louisiana	Connecticut	Louisiana
New Jersey	Mississippi	District of Columbia	Mississippi
New York	Missouri	Hawaii	Missouri
Oklahoma	North Carolina	Illinois	North Carolina
Texas	Ohio	Maine	Ohio
West Virginia	Pennsylvania	Maryland	Pennsylvania
Wisconsin	South Carolina	Montana	South Carolina
California	Tennessee	Nevada	Tennessee
Colorado	Virginia	New Mexico	Virginia
Connecticut	Wyoming	North Dakota	Wyoming
District of Columbia	Alaska	Oregon	Arizona
Hawaii	Arkansas	Rhode Island	Florida
Illinois	Delaware	South Dakota	Iowa
Maine	New Hampshire	Utah	Massachusetts
Maryland		Vermont	Michigan
Montana		Washington	Minnesota
Nevada			Nebraska

© 2020 Alberto et al. *JAMA Network Open*.

Integration		Criminalization	
**High Integration**	**Low Integration**	**Low Criminalization**	**High Criminalization**
New Mexico			New Jersey
North Dakota			New York
Oregon			Oklahoma
Rhode Island			Texas
South Dakota			West Virginia
Utah			Wisconsin
Vermont			
Washington			
Source: Young M-EDT, Wallace SP. Included, but Deportable: A New Public Health Approach to Policies That Criminalize and Integrate Immigrants. American Journal of Public Health. 2019;109(9):1171-1176.			

© 2020 Alberto et al. *JAMA Network Open*.

eTable 4: Odds ratios from logistic regression estimation of youth uninsurance by Latina maternal citizenship status and state immigrant policy environments.		
	**Youth Uninsurance (Model 1)**	**Youth Uninsurance (Model 2)**
	**Odds Ratio [95% CI]**	**Odds Ratio [95% CI]**
**Predisposing Factors**		
Maternal Citizenship Status		
US Citizen	REF.	REF.
Noncitizen	1.35*** [1.21-1.51]	1.27** [1.03-1.58]
State Immigration Policy Integration		
High Integration	REF.	
Low Integration	0.68 [0.45-1.04]	
Noncitizen*Low Integration	1.30* [1.01-1.66]	
State Immigration Policy Criminalization		
Low Criminalization		REF.
High Criminalization		1.02 [0.66-1.58]
Noncitizen*High Criminalization		1.12 [0.89-1.43]
Maternal Language		
English	REF.	
Spanish	1.18 [0.98-1.43]	1.20 [0.99-1.45]
Maternal Marital Status		
Married	REF.	
Divorced/Separated	0.79***	0.80***

© 2020 Alberto et al. *JAMA Network Open*.

	[0.69-0.91]	[0.70-0.91]
	**Youth Uninsurance (Model 1)**	**Youth Uninsurance (Model 2)**
	**Odds Ratio [95% CI]**	**Odds Ratio [95% CI]**
Never Married	0.88*** [0.82-0.93]	0.87*** [0.82-0.94]
Youth Age		
0-4	REF.	REF.
5-9	1.13** [1.05-1.22]	1.13** [1.04-1.21]
10-13	1.37*** [1.29-1.46]	1.36*** [1.28-1.45]
14-17	1.52*** [1.44-1.61]	1.52*** [1.43-1.61]
Youth Sex		
Female	0.99 [0.95-1.02]	0.99 [0.95-1.03]
Maternal Age		
18-29	REF.	REF.
30-39	0.97 [0.89-1.05]	0.97 [0.90-1.05]
40-49	1.06 [0.97-1.14]	1.05 [0.97-1.15]
50-64	0.94 [0.83-1.07]	0.94 [0.83-1.08]
Maternal Number of Youth		
1-3	REF.	REF.
4 or more	0.71*** [0.64-0.78]	0.71*** [0.65-0.79]
Maternal Employment		
Working/With a job	REF.	REF.
Unemployed	1.02	1.02

© 2020 Alberto et al. *JAMA Network Open*.

	[0.83-1.25]	[0.83-1.25]
	**Youth Uninsurance (Model 1)**	**Youth Uninsurance (Model 2)**
	**Odds Ratio [95% CI]**	**Odds Ratio [95% CI]**
Not in Labor Force	1.08 [0.99-1.17]	1.09* [1.01-1.17]
Maternal Education Level		
College Degree or more	REF.	REF.
High School Degree	1.55*** [1.37-1.76]	1.55*** [1.38-1.74]
Less than a High School Degree	1.69*** [1.43-1.99]	1.67*** [1.43-1.96]
**Enabling Factor**		
Income (%FPL)		
400% and above	REF.	REF.
300-399%	1.51*** [1.33-1.72]	1.51*** [1.32-1.70]
200-299%	1.84*** [1.65-2.05]	1.83*** [1.64-2.04]
100-199%	1.50*** [1.31-1.72]	1.48*** [1.29-1.71]
99% and below	1.20 [0.92-1.56]	1.18 [0.91-1.55]
**Contextual Characteristics**		
2016 Electoral College Result		
Clinton (Democrat)	REF.	REF.
Trump (Republican)	2.91*** [2.06-4.10]	2.60*** [1.58-4.28]
Survey Year		
2016	REF.	REF.
2017	1.04 [0.97-1.11]	1.03 [0.96-1.12]

© 2020 Alberto et al. *JAMA Network Open*.

2018	1.16**<br>[1.06-1.27]	1.16**<br>[1.05-1.27

CI, confidence interval; REF., reference.
*P<0.05.
**P<0.01.
***P<0.001.
Source: Integrated Public Use Microdata Series (IPUMS) American Community Survey, 2016-2018, N = 226,691

© 2020 Alberto et al. *JAMA Network Open*.

eTable 5: Predicted probability of youth uninsurance by Latina maternal citizenship status and integration state-level immigrant policy context.	
	**Youth Uninsurance**
	**Percentage (95% CI)**
Maternal Citizenship and Integration	
US Citizen/Low Integration	0.049*** (0.024-0.075)
US Citizen/High Integration	0.050*** (0.035-0.065)
Noncitizen/Low Integration	0.061*** (0.028-0.095)
Noncitizen/High Integration	0.083*** (0.067-0.099)

Models adjusted for youth age in years, youth sex, maternal language, maternal marital status, maternal age in years, number of youths by the mother, maternal employment status, maternal education level, income, results from 2016 Presidential election, survey year, state.
CI, confidence interval; REF., reference.
*P<0.05.
**P<0.01.
***P<0.001.
Source: Integrated Public Use Microdata Series (IPUMS) American Community Survey, 2016-2018, N = 226,691

© 2020 Alberto et al. *JAMA Network Open*.

**eTable 6: Predicted probability of youth uninsurance by Latina maternal citizenship status and criminalization state-level immigrant policy context.**	
	**Youth Uninsurance**
	**Percentage (95% CI)**
Maternal Citizenship and Criminalization	
US Citizen/Low Criminalization	0.029*** (0.025-0.035)
US Citizen/High Criminalization	0.063*** (0.039-0.086)
Noncitizen/Low Criminalization	0.037*** (0.025-0.049)
Noncitizen/High Criminalization	0.087*** (0.062-0.112)

Models adjusted for youth age in years, youth sex, maternal language, maternal marital status, maternal age in years, number of youths by the mother, maternal employment status, maternal education level, income, results from 2016 Presidential election, survey year, state.
CI, confidence interval; REF., reference.
*P<0.05.
**P<0.01.
***P<0.001.
Source: Integrated Public Use Microdata Series (IPUMS) American Community Survey, 2016-2018, N = 226,691

© 2020 Alberto et al. *JAMA Network Open*.

eTable 7: Predicted probabilities and pairwise comparison of predicted probabilities of youth uninsurance by Latina maternal citizenship status and state immigrant policy environments, excluding states who covered prenatal care regardless of documentation status.

	Youth Uninsurance
	**Percentage (95% CI)**
**Predicted Probabilities within Integration**<br>Maternal Citizenship	
US Citizen	REF.
Noncitizen/High Integration	0.016* (0.010-0.020)
Noncitizen/Low Integration	0.035*** (0.023-0.047)
**Pairwise Comparison**	0.019** (0.008-0.032)
**Predicted Probabilities within Criminalization**<br>Maternal Citizenship	
US Citizen	REF.
Noncitizen/Low Criminalization	0.010 (-0.009-0.018)
Noncitizen/High Criminalization	0.031*** (0.022-0.039)
**Pairwise Comparison**	0.021*** (0.014-0.028)

Models adjusted for youth age in years, youth sex, maternal language, maternal marital status, maternal age in years, number of youths by the mother, maternal employment status, maternal education level, income, results from 2016 Presidential election, survey year, state.
CI, confidence interval; REF., reference.
*P<0.05.
**P<0.01.
***P<0.001.
Source: Integrated Public Use Microdata Series (IPUMS) American Community Survey, 2016-2018, N = 70,648

© 2020 Alberto et al. *JAMA Network Open*.

**eTable 8: Predicted probabilities and pairwise comparison of youth uninsurance by Latina maternal citizenship status and 4-level state-level immigrant policy measure that combined integration and criminalization.**	
	**Youth Uninsurance**
	**Percentage (95% CI)**
**Predicted Probabilities**<br>Maternal Citizenship	
US Citizen	REF.
Noncitizen/High Integration, Low Criminalization	0.009** (0.003-0.017)
Noncitizen/High Integration, High Criminalization	0.023*** (0.014-0.031)
Noncitizen/Low Integration, Low Criminalization	0.069*** (0.037-0.101)
Noncitizen/Low Integration, High Criminalization	0.040*** (0.025-0.054)
**Pairwise Comparisons**<br>Maternal Citizenship	
US Citizen	REF.
Noncitizen<br>High Integration, High Criminalization vs. High Integration, Low Criminalization	0.014* (0.006-0.028)
Noncitizen<br>Low Integration, Low Criminalization vs. High Integration, Low Criminalization	0.059*** (0.026-0.092)
Noncitizen<br>Low Integration, High Criminalization vs. High Integration, Low Criminalization	0.031*** (0.014-0.040)
Noncitizen<br>Low Integration, Low Criminalization vs. High Integration, High Criminalization	0.046*** (0.030-0.052)

© 2020 Alberto et al. *JAMA Network Open*.

	Youth Uninsurance
	**Percentage (95% CI)**
Noncitizen<br>Low Integration, High Criminalization vs. High Integration, High Criminalization	0.017 (-0.003-0.027)
Noncitizen<br>Low Integration, High Criminalization vs. Low Integration, Low Criminalization	-0.029 (-0.09-0.038)
Models adjusted for youth age in years, youth sex, maternal language, maternal marital status, maternal age in years, number of youths by the mother, maternal employment status, maternal education level, income, results from 2016 Presidential election, survey year, state.<br>CI, confidence interval; REF., reference.<br>**P*<0.05.<br>***P*<0.01.<br>****P*<0.001.<br>*Source*: Integrated Public Use Microdata Series (IPUMS) American Community Survey, 2016-2018, N = 226,691	

© 2020 Alberto et al. *JAMA Network Open*.

**eTable 9: Predicted probabilities of youth uninsurance by Latina maternal citizenship status and continuous integration state-level immigrant policy context summation.**	
	**Youth Uninsurance**
	**Percentage (95% CI)**
**Predicted Probabilities**<br>Maternal Citizenship	
US Citizen	REF.
Noncitizen/2	0.043** (0.019-0.067)
Noncitizen/3	0.025*** (0.016-0.035)
Noncitizen/4	0.058*** (0.040-0.077)
Noncitizen/5	0.021*** (0.010-0.032)
Noncitizen/6	0.022*** (0.015-0.029)
Noncitizen/7	0.009 (-0.018-0.036)
Noncitizen/8	0.019* (0.004-0.034)
Noncitizen/9	-0.038*** (-0.042- -0.034)
Noncitizen/11	0.001** (0.0003-0.002)
Noncitizen/12	0.002*** (0.001-0.003)

Models adjusted for youth age in years, youth sex, maternal language, maternal marital status, maternal age in years, number of youths by the mother, maternal employment status, maternal education level, income, results from 2016 Presidential election, survey year, state.
CI, confidence interval; REF., reference.
**P*<0.05.
***P*<0.01.
****P*<0.001.
Source: Integrated Public Use Microdata Series (IPUMS) American Community Survey, 2016-2018, N = 226,691

© 2020 Alberto et al. *JAMA Network Open*.

**eTable 10: Predicted probabilities of youth uninsurance by Latina maternal citizenship status and continuous criminalization state-level immigrant policy context.**	
	**Youth Uninsurance**
	**Percentage (95% CI)**
**Predicted Probabilities**<br>Maternal Citizenship	
US Citizen	REF.
Noncitizen/1	0.003 (-0.000-0.006)
Noncitizen/2	0.014 (-0.012-0.040)
Noncitizen/3	0.026** (0.009-0.044)
Noncitizen/4	0.024*** (0.011-0.038)
Noncitizen/5	0.029*** (0.023-0.034)
Noncitizen/6	0.007 (-0.020-0.019)

Models adjusted for youth age in years, youth sex, maternal language, maternal marital status, maternal age in years, number of youths by the mother, maternal employment status, maternal education level, income, results from 2016 Presidential election, survey year, state.
CI, confidence interval; REF., reference.
**P*<0.05.
***P*<0.01.
****P*<0.001.
Source: Integrated Public Use Microdata Series (IPUMS) American Community Survey, 2016-2018, N = 226,691
